# Multifactorial predictors of falls in older adults: a decade of data from the National Health and Aging Trends Study

**DOI:** 10.1186/s12877-025-06515-2

**Published:** 2025-11-25

**Authors:** Lou C. Kohler-Voinov, Zahra N. Sayyid, Kathleen E. Cullen

**Affiliations:** 1 Department of Biomedical Engineering, Tsinghua University, Beijing, 100084 China; 2 Department of Otolaryngology-Head and Neck Surgery, Johns Hopkins University School of Medicine, Baltimore, MD 21205 USA; 3 Department of Biomedical Engineering, Johns Hopkins University School of Medicine, Traylor 501, Baltimore, MD 21205 USA; 4 Department of Neuroscience, Johns Hopkins University School of Medicine, Baltimore, MD 21205 USA; 5 Kavli Neuroscience Discovery Institute, Johns Hopkins University, Baltimore, MD 21218 USA

**Keywords:** Falls, Older adults, Fall risk factors, NHATS, Multivariable modeling, Functional status, Population-based analysis, Activities of daily living (ADLs)

## Abstract

**Background:**

Falls are a leading cause of injury and loss of independence among older adults, yet comprehensive, population-level models that integrate diverse risk factors across broad demographic groups remain limited. Prior studies often focus on isolated variables or narrow subpopulations, limiting their generalizability.

**Methods:**

To address this, we developed a robust, comprehensive model of fall risk among community-dwelling older adults using 11 years of data from the National Health and Aging Trends Study (NHATS), a longitudinal study of older adults in the United States designed to be nationally representative across a wide range of demographic and socioeconomic backgrounds. We conducted a retrospective analysis of 5,816 person-year observations from 2011 to 2022, applying univariate chi-squared tests and multivariable logistic regression to identify features associated with self-reported falls within a given month in the preceding year. Risk factors examined included sociodemographic characteristics, health status, cognitive function, and physical performance.

**Results:**

Approximately 10% of respondents reported a fall during a specific time within the past year. Consistent features associated with increased fall risk included prior fall history, impaired balance, depressive symptoms, and use of mobility aids. Cross-category analyses revealed important variations in risk profiles by age, functional status and ability to perform certain exercises.

**Conclusions:**

This study presents a decade-spanning model that reflects the multifactorial nature of fall risk and the diversity of aging trajectories in the U.S., providing a foundation for more inclusive and personalized fall prevention strategies.

**Supplementary Information:**

The online version contains supplementary material available at 10.1186/s12877-025-06515-2.

## Introduction

Falls among older adults are a growing public health concern, contributing to substantial morbidity, mortality, and healthcare costs worldwide. They are the second leading cause of injury-related deaths in this population and account for 20–30% of moderate to severe injuries and over half of fall-related hospitalizations [30]. In the United States, nearly one-third of community-dwelling adults aged 65 and older—approximately 14 million individuals—report at least one fall annually, with 37% resulting in injuries requiring medical treatment [41, 58]. Common outcomes include fractures, head trauma, hemorrhages, and persistent pain [36, 55] along with psychological and functional consequences such as depression, reduced activity, and loss of autonomy [32, 85].

Age-related declines in strength, coordination, and postural control [38, 53, 59, 64, 71, 88], compounded by cognitive impairment and multimorbidity [19, 35, 70, 86], further increase fall risk. Even low-impact falls can lead to long-term disability, institutionalization, or death—for example, one in three adults over 50 was reported to die within a year of hip fracture in earlier studies [64]. Although recent advances in care have led to modest improvements, one-year mortality rates remain alarmingly high at approximately 20–30% [7, 27, 29]. Falls are also a major source of anxiety and psychological stress [6, 15, 35, 37, 50]. With older adults comprising an increasing share of the global population [92], prevention efforts are increasingly urgent.

Accurate risk assessment is central to fall prevention. Although many studies have identified key contributors—such as fall history [45, 46, 72, 74, 93], impaired balance [3, 25, 33, 46, 57, 74], vestibular and visual deficits [47, 74, 75, 94], cognitive [24] and nutritional [2] difficulties, hearing loss [90], purpose in life [80], and chronic health conditions [39, 44, 74, 76, 93]—many models are derived from small or cross-sectional samples, limiting generalizability [40, 48, 66, 66, 83]. Early population-based studies generally considered relatively narrow set of variables [12, 17, 20, 26, 28, 62, 78, 84], and even more recent work focused on large cohorts [1, 31, 82, 87, 91] has continued to work with a limited number of variables.

The National Health and Aging Trends Study [60] offers a uniquely powerful resource to address these limitations. This nationally representative longitudinal study of older U.S. adults includes detailed annual assessments spanning physical health, functional status, healthcare use, psychosocial well-being, and social and environmental determinants, as well as fall history. To date, however, no model has comprehensively leveraged the breadth of NHATS to assess fall risk across demographic and functional groups. Accordingly, in this study, we analyzed 11 years of NHATS data (2011–2022), comprising 5,816 person-year observations, to identify features associated with falling among community-dwelling older adults. Our analysis incorporated 94 variables across sociodemographic, clinical, psychological, and functional domains. Using univariate and multivariable modeling, we identified key risk factors and explored subgroup differences by age, functional status and ability to perform certain exercises. These findings provide a comprehensive foundation for improving fall risk stratification and guiding targeted prevention strategies.

## Methods

The NHATS is a large-scale cohort study of 8500 Medicare beneficiaries of ages 65 and older. This ongoing study collects data from participants via an annual in-person interview, including information on the participant’s physical and cognitive capacities, how certain activities of daily life are carried out, the social, physical, and technological aspects of the participant’s environment, and their participation in certain activities. Moreover, the study conducts a series of performance-based tests providing complimentary measures of the participant’s physical and cognitive capacities. Finally, information is obtained on living arrangements, economic status and well-being, and aspects of early life.

**Fig. 1:**
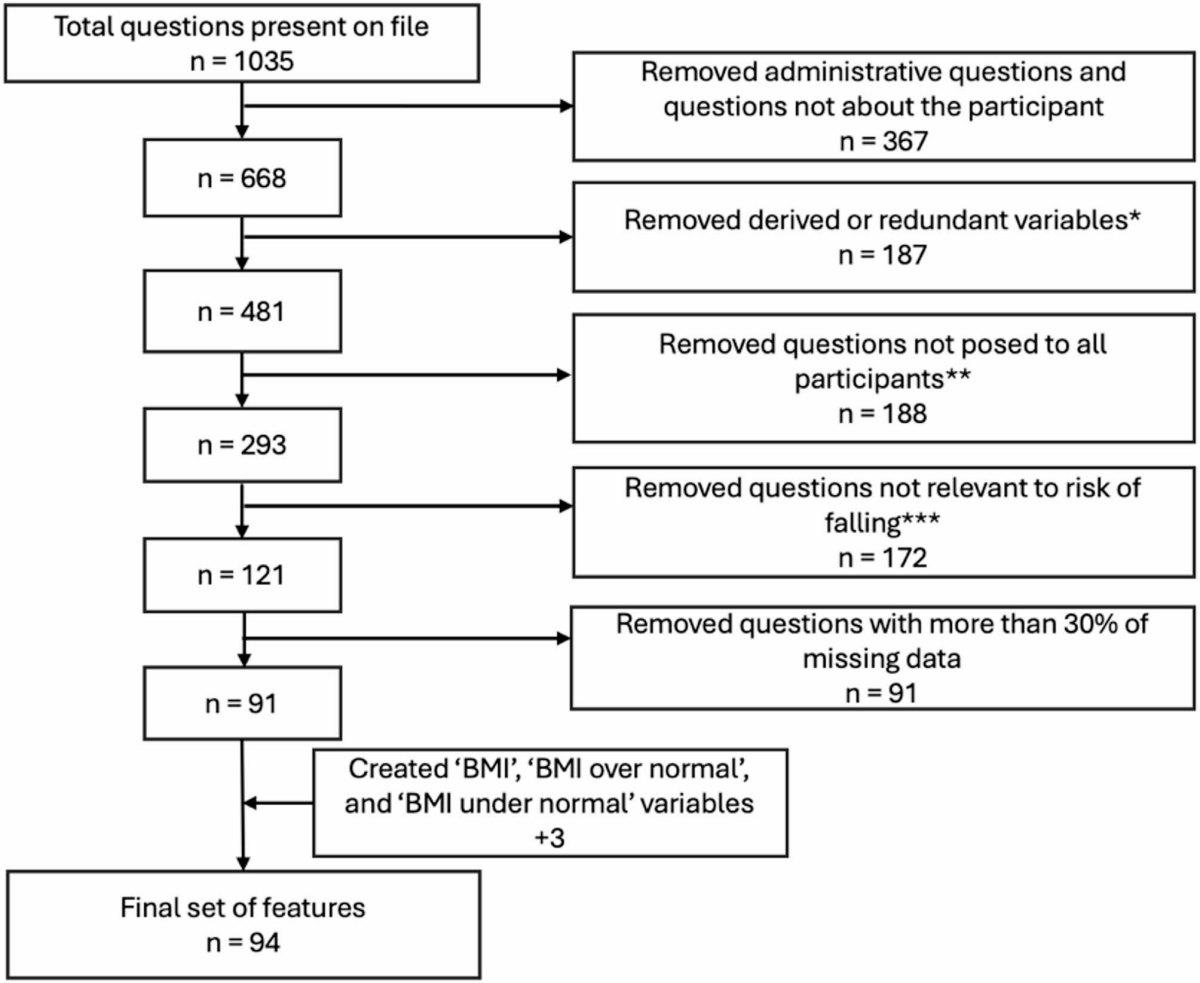
Feature selection process. *n* = Number of features. *The NHATS recorded responses included questions with similar meanings but different phrasing, which we qualified as redundant and variables which summarized each section, which we termed derived. **Some additional questions were sometimes asked to participants based on their previous answers, such as to get additional details. Since these were not asked to all participants, these were removed from our set of features. ***Questions were not considered relevant if another variable already encompassed the information from other questions. Questions were considered relevant if they were related to the participant’s health situation, their mobility and capabilities, and selected summary variables about family situation and community, residence physical structure, and socio-economic status

The collected data comprised answers from each participant to the questions posed by an interviewer in the form of multiple choice. From the initial set of questions, 94 of these were selected to serve as features to use in a logistic regression model, selected to cover a wide range of categories from the interview (e.g. Health Conditions, Household, Physical Capacity) and noted as relevant in the current literature (Fig. 1). A comprehensive list of these features can be found in the Supplemental Materials (Supp Table 1). In preprocessing the data, all participants who had missing responses for any of the 94 features used in the model were removed. Since the NHATS is a longitudinal study and new participants are added to the study each year, participants were incorporated into our overall data across the years as independent instances. Participants who were initially interviewed between the beginning of 2012 and the end of 2022 (Supp Table 2) were incorporated, for a total of 5,816 participants. The purpose of this investigation was to determine and investigate risk factors associated with falling during the month one year after the initial interview. For each participant, answers were recorded in one year, as well as the year immediately following. Answers from the first year were used as features, and this work investigated the link between them and their response in the following year to the question: “Have you fallen in the last month?“. Thus, the outcome variable is a binary “yes” or “no” response to this question.

Conditionally administered items (Fig. 1) were identified from the publicly available NHATS Data Collection Instruments. These items were excluded to ensure that all participants answered an identical set of questions. For instance, vaccine-related follow-up questions were only presented to respondents who reported prior vaccination. Variable identification and exclusion were performed by L.C.K.V. and reviewed by the senior authors. In most cases, excluded items were derived or summary variables already captured by earlier questions. Performance-based assessments were administered and scored by trained NHATS interviewers, typically in participants’ residences or assisted living facilities. Further details on data collection procedures are available on the NHATS database website.

### Statistical analysis

Chi-squared tests were performed on all possible entries for all features and reported on all those that showed statistical significance (P *<* 0.05) in Tables 1, 2 and 3. The results for all features can be found in Supplemental Materials (Supp Table 1). Since this study is exploratory, and we are investigating the presence of risk factors, we did not perform corrections for multiple comparisons for the univariate chi-squared tests. To examine the effect of each category within the category variables, a chi-squared test was performed between each category and the one above. For example, for age, the age bracket “65–69” was compared to the age bracket “70–74”, and the age bracket “70–74” was compared to the age bracket “75–79”. For these category comparisons, correction for multiple comparisons was employed when evaluating significance. All such comparisons can be found in Supplementary Materials (Supp Tables 3, 4, 5, 6 and 7). For non-categorical variables, a t-test for independence was conducted.

**Table 1:** Population characteristics (total cohort): overall demographic information

Risk factor	Total cohort (*N* = 5816)	Did not fall (*N* = 5251)	Fell (*N* = 565)	*P*
Age (years) ^R^				< 0.001*
65–69	1088 (18.7%)	1022 (19.5%)	66 (11.7%)	
70–74	1483 (25.5%)	1357 (25.8%)	126 (22.3%)	
75–79	1288 (22.1%)	1151 (21.9%)	137 (24.2%)	
80–84	1075 (18.4%)	942 (17.9%)	133 (23.5%)	
85–90	627 (10.7%)	561 (10.6%)	66 (11.7%)	
90+	255 (4.4%)	218 (4.5%)	37 (6.5%)	
Overall health condition				< 0.001*
Excellent	793 (13.6%)	740 (14.1%)	53 (9.3%)	
Very good	1926 (33.1%)	1775 (33.8%)	151 (26.7%)	
Good	1990 (34.2%)	1804 (34.4%)	186 (32.9%)	
Fair	907 (15.5%)	769 (14.6%)	138 (24.4%)	
Poor	200 (3.3%)	163 (3.1%)	37 (6.5%)	
Comorbidities and objective health issues				
Heart disease^1^	1050 (18.0%)	916 (17.4%)	134 (23.7%)	< 0.001*
High blood pressure^1^	3977 (68.5%)	3565 (67.9%)	412 (72.9%)	0.016*
Arthritis^1^	3440 (59.2%)	3054 (58.2%)	386 (68.3%)	< 0.001*
Diabetes^1^	1518 (26.0%)	1338 (25.5%)	180 (31.8%)	0.001*
Lung disease^1^	962 (16.5%)	833 (15.9%)	129 (22.8%)	< 0.001*
Stroke^1^	106 (1.7%)	89 (1.7%)	17 (3.0%)	0.040*
Hearing problems	1268 (21.8%)	1107 (21.1%)	161 (28.4%)	< 0.001*
History of broken or fractured bones^1^	241 (4.0%)	203 (3.8%)	38 (3.9%)	0.002*
Fell in the last year ^R^	1402 (24.0%)	1176 (22.4%)	226 (40.0%)	< 0.001*
Overnight hospital stay^1^	1166 (20.0%)	1021 (19.4%)	145 (25.7%)	< 0.001*
Lost 10 pounds^1 R^	1470 (25.2%)	1284 (24.4%)	186 (32.9%)	< 0.001*
Subjective health issues				
Problems with chewing or swallowing^2^	440 (7.5%)	370 (7.0%)	70 (12.4%)	< 0.001*
Have problems speaking^2^	198 (3.3%)	162 (3.1%)	36 (6.4%)	< 0.001*
Bothered by pain^2^	3055 (52.6%)	2705 (51.5%)	350 (61.9%)	< 0.001*
Have breathing problems^2^	198 (3.3%)	162 (3.1%)	36 (6.4%)	< 0.001*
Limited strength in the body^2 R^	2514 (43.2%)	2181 (41.5%)	333 (58.9%)	< 0.001*
Low energy^2^	2421 (41.7%)	2117 (40.3%)	304 (53.8%)	< 0.001*
Balance and coordination problems^2 R^	1528 (26.3%)	1278 (24.3%)	250 (44.2%)	< 0.001*
Worried about falling^2^	1512 (26.0%)	1282 (24.4%)	230 (40.7%)	< 0.001*
Use medication for pain^2^				< 0.001*
Never	2084 (35.9%)	1897 (36.1%)	187 (33.1%)	
Rarely (once a week or less)	1438 (24.7%)	1330 (25.3%)	108 (19.1%)	
Some days (2–4 a week)	1023 (17.5%)	912 (17.4%)	111 (19.6%)	
Most days (5–6 a week)	289 (4.9%)	249 (4.7%)	40 (7.1%)	
Every day	982 (16.8%)	863 (16.4%)	119 (21.1%)	
Depressive, anxious, or unsatisfied traits^2 R^				< 0.001*
Not at all	2784 (47.9%)	2577 (49.0%)	207 (36.6%)	
Several days	2016 (34.8%)	1804 (34.3%)	212 (37.5%)	
More than half the days	484 (34.9%)	410 (7.8%)	74 (13.1%)	
Nearly every day	532 (9.0%)	460 (8.8%)	72 (12.7%)	

For a graphical representation of the interview timeline, the reader is encouraged to consult Supp Fig. 1

^1^ The question refers to the period of the year prior to the interview

^2^ The question refers to the period of the month prior to the interview

^R^ This risk factor was found to be significant in the logistic regression (Table 4)

*P *<* 0.05

**Table 2:** Population characteristics (total cohort): activities of daily living (ADL)

Risk factor	Total cohort (*N* = 5816)	Did not fall (*N* = 5251)	Fell (*N* = 565)	*P*
Transferring				
How often holds onto walls or furniture^2^				< 0.001*
Never	3242 (55.8%)	3014 (57.4%)	228 (40.3%)	
Rarely	1298 (22.3%)	1167 (22.2%)	131 (23.2%)	
Sometimes	911 (15.6%)	774 (14.7%)	137 (24.2%)	
Most times	229 (3.8%)	194 (3.7%)	35 (6.2%)	
Every time	136 (2.2%)	102 (1.9%)	34 (6.0%)	
Difficulty getting out of bed^2^				< 0.001*
None	5039 (86.9%)	4588 (87.4%)	451 (78.8%)	
A little	518 (8.8%)	443 (8.4%)	75 (13.3%)	
Some	219 (3.7%)	188 (3.5%)	31 (5.5%)	
A lot	40 (0.6%)	32 (0.6%)	8 (1.4%)	
Can carry 20 pounds^2^	4434 (76.4%)	4066 (77.4%)	368 (65.1%)	< 0.001*
Able to get on knees and back up^2^	2697 (33.5%)	2507 (47.7%)	190 (33.6%)	< 0.001*
Able to put a heavy object on a shelf^2^	4979 (85.8%)	4547 (86.6%)	432 (76.5%)	< 0.001*
Ambulating				
Uses a cane, walker, wheelchair, or scooter^2^	1234 (21.2%)	1036 (19.7%)	198 (35.0%)	< 0.001*
Used help to get outside^2^	258 (4.3%)	211 (4.0%)	47 (8.3%)	< 0.001*
How often leaves their building vs. a year ago				< 0.001*
Less often	578 (9.9%)	495 (9.4%)	83 (14.7%)	
About the same	4762 (82.1%)	4319 (82.2%)	443 (78.4%)	
More often	476 (8.1%)	437 (8.3%)	39 (7.0%)	
Difficulty in going outside^2 R^				< 0.001*
None	5182 (89.3%)	4741 (90.3%)	441 (78%)	
A little	406 (6.9%)	327 (6.2%)	79 (14.0%)	
Some	181 (3.0%)	147 (2.3%)	34 (6.0%)	
A lot	47 (0.7%)	36 (0.7%)	11 (2.0%)	
Able to walk 6 blocks^2^	4030 (69.4%)	3722 (71.0%)	308 (54.5%)	< 0.001*
Can walk up 20 stairs^2^	4462 (76.9%)	4105 (78.2%)	357 (63.2%)	< 0.001*
How often goes outside^2^				0.037*
Never	0 (0%)	0 (0%)	0 (0%)	
Rarely (once a week or less)	109 (1.8%)	95 (1.8%)	14 (2.5%)	
Some days (2–4 a week)	529 (9.0%)	462 (8.8%)	67 (11.8%)	
Most days (5–6 a week)	1008 (17.3%)	923 (17.6%)	85 (15.0%)	
Every day	4170 (71.9%)	3771 (71.8%)	399 (70.6%)	
Difficulty getting around inside the house ^2^				< 0.001*
None	5157 (88.9%)	4713 (89.7%)	444 (78.6%)	
A little	459 (7.8%)	378 (7.2%)	81 (14.3%)	
Some	184 (3.1%)	149 (2.8%)	35 (6.2%)	
A lot	16 (0.2%)	11 (0.2%)	5 (0.9%)	
Toileting				
Difficulty using the toilet^2 R^				< 0.001*
None	5598 (96.5%)	5078 (96.7%)	520 (92.0%)	
A little	153 (2.5%)	118 (2.2%)	35 (6.2%)	
Some	55 (0.7%)	49 (1.0%)	6 (1.1%)	
A lot	10 (0.1%)	6 (0.1%)	4 (0.7%)	
Uses tools to aid in toilet use	1318 (22.6%)	1139 (21.7%)	179 (31.7%)	< 0.001*
Needs help using the toilet	20 (0.2%)	14 (0.2%)	6 (1.0%)	0.007*
Bathing				
Difficulty in washing up^2^				< 0.001*
None	5353 (92.3%)	4860 (92.5%)	493 (87.2%)	
A little	336 (5.6%)	284 (5.4%)	52 (9.2%)	
Some	104 (1.7%)	86 (1.6%)	18 (3.1%)	
A lot	23 (0.3%)	21 (0.4%)	2 (0.3%)	
Feeding				
Can open a jar with one hand^2^	4531 (78.0%) %)	4136 (78.8%)	395 (69.9%)	< 0.001*

^1^ The question refers to the period of the year prior to the interview

^2^ The question refers to the period of the month prior to the interview

^R^ This risk factor was found to be significant in the logistic regression (Table 4)

*P *<* 0.05

**Table 3:** Population characteristics (total cohort): scores on NHATS activities

Risk factor	Total cohort (*N* = 5816)	Did not fall (*N* = 5251)	Fell (*N* = 565)	*P*
Grip score				0.027*
0 (worst)	463 (7.9%)	411 (7.8%)	52 (9.2%)	
1	1256 (21.6%)	1120 (21.3%)	136 (24.1%)	
2	1398 (24.0%)	1260 (24.0%)	138 (24.4%)	
3	1409 (24.2%)	1266 (24.1%)	143 (25.3%)	
4 (best)	1290 (22.1%)	1194 (22.7%)	96 (17.0%)	
Balance score				< 0.001*
0 (worst)	271 (4.6%)	234 (4.4%)	37 (6.5%)	
1	1126 (19.3%)	972 (18.5%)	154 (27.2%)	
2	1608 (27.6%)	1433 (27.3%)	175 (31.0%)	
3	1453 (25.0%)	1321 (25.1%)	132 (23.3%)	
4 (best)	1358 (23.3%)	1291 (24.6%)	67 (11.9%)	
Walking score				< 0.001*
0 (worst)	201 (3.4%)	173 (3.3%)	28 (5.0%)	
1	1399 (24.0%)	1217 (23.2%)	182 (32.2%)	
2	1508 (25.9%)	1354 (25.8%)	102 (27.3%)	
3	1377 (23.7%)	1275 (24.3%)	154 (18.0%)	
4 (best)	1331 (22.9%)	1232 (23.5%)	99 (17.5%)	
Chair score				< 0.001*
0 (worst)	830 (14.2%)	718 (13.7%)	112 (19.8%)	
1	1198 (20.6%)	1061 (20.2%)	137 (24.2%)	
2	1227 (21.1%)	1112 (21.2%)	115 (20.3%)	
3	1349 (23.2%)	1241 (23.6%)	108 (19.1%)	
4 (best)	1212 (20.8%)	1119 (21.3%)	93 (16.4%)	

^1^ The question refers to the period of the year prior to the interview

^2^ The question refers to the period of the month prior to the interview

^R^ This risk factor was found to be significant in the logistic regression (Table 4)

*P *<* 0.05

**Table 4:** Significant risk factors in the logistic regression associated with falls during the month 11 months after the initial interview

Risk factor	OR	CI 95%
Demographic data		
Age		
75 to 79	1.60	1.08–2.36
80 to 85	1.87	1.24–2.82
90+	1.99	1.08–3.65
Information adjacent to health		
Fallen in the past year	1.94	1.53–2.46
Down/depressed/hopeless: Nearly every day	0.32	0.12–0.86
Lost 10 pounds	1.30	1.02–1.66
Takes medication for sleep	1.54	1.04–2.27
Information on home situation		
Total number in household	1.15	1.04–1.27
Information on mobility and general ability		
Limited strength in the lower body	1.47	1.13–1.90
Balance and coordination problems	1.46	1.12 −1-91
Difficulty in going outside: A little	1.66	1.11–2.48
How often holds onto walls/furniture: Every time	1.89	1.02–3.49
Ability to perform daily activities		
Difficulty using the toilet: A little	2.57	1.46–4.53
Difficulty using the toilet: A lot	23.70	2.86 −196.52

Logistic regression was performed between all our 94 features and whether the person had fallen the following year. This model was built to account for the multifactorial nature of fall risk. For each feature, odds ratios and confidence intervals were computed. No regularization was used in the training of this model, however comparisons in performance metrics were made to a model using lasso regularization.

## Results

We employed a prospective modeling approach to assess whether older adults would experience a fall during the specific month of their follow-up NHATS interview, using features obtained one year earlier. This design enabled us to identify features associated with imminent fall risk in a naturalistic, real-world setting. Of the 5,816 participants analyzed, 565 (9.7%) reported a fall in the target follow-up window, while 5,251 (90.3%) did not. We first examined differences in population characteristics between these groups, then used logistic regression to identify the most highly associated risk factors for future falls.

### Demographic and Self-Reported Health Characteristics

Older age was associated with increased fall risk (*P* < 0.001, Table 1). However, when correcting for multiple comparisons among age categories (*P* = 0.05/5 = 0.01), no single group-wise comparison reached significance (Supp Table 3). This suggests that while fall risk increases with age at the population level, age alone may not reliably differentiate individual risk without considering other health or functional measures.

Indeed, self-rated overall health was significantly worse among individuals who experienced a future fall (*P* < 0.001). After correcting for comparisons between the four response categories (*P* = 0.0125), the only statistically significant contrast was between “Good” and “Fair” health (*P* < 0.001, Supp Table 4). Interestingly, participants reporting “Excellent” or “Poor” health did not differ significantly from other groups, suggesting that intermediate health ratings may more sensitively capture emerging declines associated with fall risk. Medication use for pain, often reflective of chronic musculoskeletal issues, was another significant factor. Compared to those who reported taking such medications “Rarely” (once per week or less), those who took them on “Some days” (2–4 days per week) were significantly more likely to fall (*P* < 0.0125). This may indicate that moderate but persistent pain management needs—potentially associated with undiagnosed functional limitations—are more highly associated with instability than either rare or frequent usage alone. Mental health measures also revealed a complex association with fall risk. Individuals reporting depressive symptoms on “Several days” per week were more likely to fall compared to those who reported no symptoms (*P* < 0.001) or symptoms on “More than half the days” (*P* = 0.007). These findings (corrected threshold *P* = 0.016) suggest moderate but consistent depressive symptoms may signal a particularly vulnerable state, potentially reflecting a combination of impaired motivation, reduced physical engagement, and early cognitive changes.

### Functional Limitations, Mobility Status, and Social Context

Indicators of mobility limitations were among the strongest univariate features associated with future falls. Use of mobility aids or reliance on another person for assistance were both highly significant (*P* < 0.001, Table 2). Similarly, the use of toileting aids—typically reserved for individuals with advanced physical limitations—was significantly more common among those who later fell (*P* < 0.001). These results emphasize the importance of direct questions about support use, which may serve as efficient proxies for fall risk in clinical or public health screening tools. Changes in mobility behavior over time also proved informative. Participants who reported leaving their building “Less often” compared to the previous year were more likely to fall than those whose activity remained “About the same” (*P* < 0.025, Supp Table 5). Notably, those who reported “Never” holding onto walls or furniture while walking were actually more likely to fall than those who did so “Rarely” (*P* < 0.0125), a counterintuitive finding that may reflect underreporting of subtle balance problems or overconfidence in mobility.

When examining difficulty in performing daily tasks, only the contrast between “none” and “a little” reached statistical significance across multiple domains (*P* < 0.001, Supp Table 6). This pattern suggests that individuals with even mild, self-perceived difficulties may already be experiencing functional declines that precede overt instability. Conversely, more severe difficulty may be associated with behavioral adaptations (e.g., reduced movement) that buffer fall risk. Several specific mobility-related abilities—such as walking six blocks, climbing 20 stairs, and kneeling and standing back up—were significantly more prevalent among those who did not fall. These abilities represent complex movements requiring strength, balance, and confidence, and their absence may serve as early warning signs of vulnerability.

Environmental and social context also appeared to influence fall risk. Participants who had not fallen lived in slightly larger households (mean 2.09 ± 1.15) than those who had fallen (mean 1.97 ± 1.02), a difference that reached statistical significance (*P* = 0.006). This association suggests that living with others may confer protective benefits—potentially through greater informal supervision, help with daily activities, or reduced social isolation. Although household size is a relatively coarse metric, it may reflect the degree of embeddedness within a supportive living environment. Unfortunately, more detailed economic indicators could not be analyzed due to insufficient reporting of wealth data across the cohort.

#### Objective Performance-based Measures

Performance on standardized physical function tests offered further discriminatory power. In chair stands, walking, and balance exercises, participants who experienced future falls were significantly more likely to have scored in the lowest categories (scores of 0 or 1, Table 3). In the NHATS balance test, scoring 3 versus 4 was the only pairwise comparison that remained significant after correction (*P* < 0.0125, Supp Table 7). These findings reinforce the utility of standardized physical assessments for identifying individuals at elevated risk—particularly when subjective self-assessments may be biased or limited.

##### Multivariate Modeling of Fall Risk

While many individual characteristics—ranging from physical performance and functional status to mental health and household structure—showed significant associations with future fall risk, these factors are often interrelated. Accordingly, to determine which features were independently associative, we conducted a multivariate logistic regression analysis. This approach allowed us to isolate the most robust features associated with falls by accounting for the broader constellation of co-occurring risk factors. We focused the model on fall incidence in the 11th month following the baseline survey (Table 4). Several variables retained significance in the multivariate analysis, including older age groups, subjective health assessments, depressive symptoms, pain medication use, mobility aid use, and performance-based indicators of physical function. Strikingly, difficulty using the toilet emerged as the most powerful single associated feature, with the highest odds ratio across all features. This finding complements our univariate results and underscores toileting difficulty—an often underrecognized and sensitive indicator—as a key marker of declining functional independence. While the cause of toileting difficulty was not specified in the original responses, its strong associative value likely reflects a convergence of impairments in strength, balance, joint mobility, and cognitive function. Additional significant factors found from this analysis were being over 90 years old, reporting holding onto furniture of walls “Every time” and having fallen in the past year.

Thus, taken together, these findings underscore the multifactorial nature of fall risk and demonstrate that both subjective reports (e.g., health perception, depressive symptoms) and objective indicators (e.g., balance tests, mobility aid use) contribute uniquely to identifying high-risk individuals. Importantly, several of the most strongly associated features—for example, toileting difficulty or pain medication use—are easily queried in clinical or public health settings and may be readily integrated into screening protocols for fall prevention.

To evaluate model performance and robustness, we compared the full logistic regression model to a reduced model containing only the features listed in Table 4. The full model yielded AIC, BIC, and McFadden R² values of 3631.37, 4631.62, and 0.10, respectively. In contrast, the reduced model produced an AIC of 3524.87 and a BIC of 3771.60, suggesting a more favorable tradeoff between goodness of fit and model complexity. However, its McFadden R² decreased to 0.07, indicating a reduction in overall fit. Thus, while the full model provided the best fit to the data, the reduced model achieved better tradeoff by balancing fit with fewer parameters. Further, although predictive accuracy was not the primary focus of this study, we report AUROC values and ROC curves for both models in Supplementary Fig. 2. Finally, to examine the impact of regularization, we trained a model with lasso penalty. This model yielded an AIC of 3553.55, BIC of 3913.64, and McFadden R² of 0.07, further supporting that the unregularized full model performed best despite its larger parameter set.

## Discussion

In this large, nationally representative study of community-dwelling older U.S. adults, we identified a wide range of features associated with fall risk by analyzing 94 variables encompassing physical health, functional capacity, and psychosocial well-being. Building on and substantially expanding earlier population-level models, our findings reaffirm the importance of established risk factors—such as advanced age, prior falls, balance impairments, and mobility limitations—while also highlighting the significance of underrecognized contributors, including subjective health ratings, depressive symptoms, and difficulties with activities of daily living. Notably, toileting difficulty emerged as the strongest single features associated with future falls, emphasizing the need to assess discrete functional limitations beyond general mobility measures. Together, these results reinforce the multifactorial nature of fall risk and demonstrate the utility of a comprehensive, data-driven approach that integrates objective performance measures with self-reported indicators. By identifying a broad spectrum of modifiable and non-modifiable risk factors, our study provides actionable insights to support more targeted, contemporary fall prevention strategies at both individual and population levels.

The detailed results reported in Tables 1, 2, 3 and 4 provide the empirical foundation for the summary findings described above. Compared to individuals who did not report a fall, those who did were significantly older and exhibited poorer health across both objective and subjective measures (Table 1). They also showed greater reliance on assistance with activities of daily living, particularly in areas such as mobility and toileting (Table 2). Additionally, they showed worse performance overall on the grip, balance, walking and chair NHATS performance activities, with higher proportions of lower scores and statistically significant differences overall (Table 3).

It has long been known that fall risk increases with age [4, 13] Shahudin et al., 73, but while there were proportionately fewer people in the youngest age group who fell, comparing increasing age groups did not show significant differences (Supp Table 3). Risk associated with age may increase too gradually to be detected by such a test. Self-reported performance in various mobility activities (e.g. using help to get outside, needing help with using the toilet, ability to walk 6 blocks) was also found to be highly significant (Table 2). These capabilities are potentially more easily quantified and could offer context for a participant’s health beyond their medical background, potentially providing valuable information on fall risk. Similarly, even minor difficulties in daily activities were linked to a higher likelihood of falling (Supp Table 5). General mobility questions may thus serve as useful indicators, as opposed to more traditional tools like the Timed Up and Go test [69], which has faced scrutiny in predicting falls [11, 16, 64]. Previous falls, the use of assistive devices, and acute or chronic illness have been previously linked to falls [34, 67, 81], findings we confirm here. Impaired balance and gait, previously known contributors to reduced mobility and falls [18, 21, 63, 64], were also significant in our analysis, supporting balance and walking performance as potential fall screening tools. This finding is particularly relevant as other assessments, such as the Elderly Mobility Scale [77], Hierarchical Assessment of Balance and Mobility [51, 52] and the Physical Performance Mobility Examination [89], have demonstrated limitations in effectively assessing mobility in older adults [22]. We speculate that age-related declines in somatosensory, visual, and vestibular function, which are essential for postural control [23] may contribute to impaired balance and mobility. However, these specific factors were not captured in the NHATS data.

While univariate analyses identified a broad range of associated factors, multivariate logistic regression (Table 4) isolated a smaller set of independent features. These included prior falls, age, and recent weight loss, as well as subjective reports of limited lower-body strength, balance and coordination issues, and frequent depressive symptoms. Among ADL-related variables, difficulty with toileting and reduced ability to leave the home emerged as the strongest features associated with future falls. These results reinforce the multifactorial and multidimensional nature of fall risk and clarify which specific factors carry the greatest associative weight within a large, representative sample. Interestingly, a participant’s description of their own general health was found to be highly significant (Table 4). This self-reported marker has inherent limitations, as it only offers a broad, subjective assessment of health. Nonetheless, it is encouraging that it remains significant in evaluating fall risk.

Population-level approaches to fall risk have been commonly used to date [12, 20, 31, 49, 58, 62, 91], and remain highly relevant given the aging population and growing need for scalable prevention strategies. Recently, Moreland et al. [58] examined a U.S. cohort (*N* = 142,591) using a limited set of 10 features. While the cohort demographic was very similar, the authors focused on observing trends in 10 fall-related features, comparing percentages across the years from 2012 to 2018. Building on this foundation, our study incorporates a more comprehensive set of 94 features spanning physical, functional, and psychosocial domains. Despite these methodological differences, their findings that (i) falls were more common among individuals in poorer health and (ii) those with limitations in activities of daily living (ADLs) align with ours. Moreover, our findings extend these results by showing that fall risk is also significantly influenced by subjective health assessments, psychological well-being, and environmental factors - most notably self-perception of general health, strength and balance ability, as well as the number of people present in the household, highlighting the need for multifactorial screening approaches that go beyond traditional clinical indicators.

Interestingly, our analysis of a U.S. cohort aligns closely with findings from recent international studies involving similarly sized populations, including the English Longitudinal Study of Aging [31] (*N* = 4,301) and the Chinese Longitudinal Survey on Urban and Rural Elderly [91] (*N* = 16,393), compared to *N* = 5,816 in the present study. While those studies examined fewer features (17 and 28, respectively), we found strong agreement across overlapping variables. Age emerged as a consistent feature associated with falls across all three studies. The English study, like ours, found that BMI was not a significant feature and that physical activity was protective—though only among men. Similarly, the Chinese study reported associations with heart disease, arthritis, self-rated health, ADL disability, and depression, mirroring our own findings. Interestingly, while cognitive impairment was a significant factor in both international studies—assessed via memory-related questions in the Chinese study and tests of verbal/prospective memory, attention, and executive function in the English study—our measure of memory decline was not. Importantly, the broader range of features in our study allowed us to identify additional features associated with falls not previously reported, including sleep disturbances, performance on a battery of tests, and sensory and mobility impairments. Overall, despite examining a broader set of features, our results are largely consistent with findings from other countries, reinforcing the robustness of shared risk factors across diverse aging populations.

The significant factors identified in our study underscore the complex nature of fall risk in older adults. Although features were assessed prior to fall occurrence, our design could not control for confounding factors. For instance, both fear of falling and past-year falls were significant features; however, prior falls may drive fear, making it unclear whether fear alone is associated with future risk. As shown previously, fear often leads to activity avoidance and deconditioning, which increase fall risk [33]. Conversely, confidence in mobility—like high self-rated health—may reflect lower risk. Fall risk involves both modifiable and non-modifiable factors, with the former providing opportunities for prevention. Univariate analyses identified a wide range of relevant features (Tables 1, 2 and 3), while logistic regression highlighted a smaller set—age, past-year falls, balance or coordination problems, and toileting difficulty—as the strongest associated features (Table 4). Interestingly, daily depression or anxiety was associated with reduced fall risk (Table 4, OR < 1), contrary to prior findings [61]. We also did not observe a significant gender difference in fall risk, in contrast to some prior studies reporting higher risk among men [61, 81], or women [31] see Supp Table 1). These findings align with prior evidence supporting interventions such as medication review, home safety modifications, strength and balance training, and post-fall recovery strategies [81]. Many of the key risk indicators identified here are readily observable by clinicians, caregivers, and family members, enabling earlier intervention.

Building on these findings, our results have important implications for clinical screening and prevention. Specifically, they can help inform the development of potential new tools for primary care and other providers caring for older adults. Future studies should also evaluate how the identified features might contribute to refining existing risk stratification approaches, such as the STEADI algorithm [79] and its Stay Independent Questionnaire, or be incorporated into updated clinical practice guidelines (Panel on Prevention of Falls in Older Persons, American Geriatrics Society and British Geriatrics Society 65; [68]. Integrating these features into screening protocols could enable more strategic resource allocation, tailored interventions, and improved patient outcomes while reducing costs. While several of the features we identified overlap with those in existing questionnaires—such as worry about falling, reliance on furniture for support, use of sleep medications, and history of prior falls—our study also highlights novel indicators. In particular, difficulty with toileting, lower body weakness, and balance or coordination problems may represent important additions for future screening and intervention efforts.

The dataset that formed the basis of our study relied on questionnaire data, whereas inertial measurement units and posturography are now increasingly used in fall risk detection [5, 8, 9, 14, 43, 54]. However, while these sensor-based methods are valuable, they also require more resources, specialized expertise, and additional testing time. In comparison, the NHATS questionnaire is efficient and widely deployable advantages that would increase further if streamlined to emphasize the most informative items. The risk factors identified through our approach are also likely more intuitive for clinicians to interpret. Importantly, our dataset includes responses from individuals across all U.S. states, with deliberate efforts to represent historically underrepresented groups in research [42, 56], including Black, Hispanic, and older adults. This enhances the generalizability of our findings. Finally, although we used traditional statistical methods, emerging machine learning approaches may provide complementary insights [10]. Future work could apply these techniques to assess the robustness of our findings and identify additional risk factors.

### Limitations

This study focused on falls occurring within the month preceding the follow-up interview. Although similar analyses could have been conducted using 12-month recall, we deliberately selected the 1-month measure to ensure a clear temporal separation between predictor assessment and outcome. Using a 12-month recall would have obscured event timing, since a fall occurring one week versus eleven months after baseline would be indistinguishable. By focusing on 1-month falls assessed one year later, we sought to isolate features most relevant to risk over the subsequent year. Nevertheless, this one-year gap between predictor assessment and outcome measurement may have missed intervening factors influencing fall risk. Future work could therefore compare models using both 1-month and 12-month recall to provide a more comprehensive picture. A second limitation arises from excluding participants with missing responses. While this approach introduces potential bias and limits generalizability, it allowed us to construct a dataset in which all participants completed the same set of items. Although this reduced sample size, the analytic cohort still exceeded 5,000 individuals—considered large for studies in this field.

## Conclusion

This study provides a comprehensive analysis of the NHATS dataset as it related to falls in older adults. Associations were made between events separated by one year, and risk factors were analyzed. A variety of factors were found to be statistically significant, including both objective and subjective descriptors of health as well as dependance on external aid for ADLs. This work encourages medical practitioners screening for fall risk to consider using the factors in this study, and to push for a more holistic screening approach when assessing future falls.

## Supplementary Information

Supplementary Material 1.

## Data Availability

Raw data may be downloaded from the NHATS website (https://nhats.org/researcher). Treated data and analysis from this study can be made available to other researchers upon request made to the corresponding author and with appropriate approvals. This study was not pre-registered in any databases.
