# Supplementary material for: Multifactorial predictors of falls in older adults: a decade of data from the National Health and Aging Trends Study
Source: BMC Geriatr. 2025 Nov 25;25:950. doi: 10.1186/s12877-025-06515-2 (PMC12645780; doi:10.1186/s12877-025-06515-2)
Supplement: Supplementary file 1 — Supplementary Material 1. [file 12877_2025_6515_MOESM1_ESM.docx]

# **Supplemental Materials**

Supplementary Table 1: Population characteristics for entire dataset (total cohort).

| Risk factor | Total cohort (N=5816) | Did not fall (N=5251) | Fell (N=565) | P |
| --- | --- | --- | --- | --- |
| **Demographic data** |  |  |  |  |
| Age (years) |  |  |  | <.001* |
| 65-69 | 1088 (18.7%) | 1022 (19.5%) | 66 (11.7%) |  |
| 70-74 | 1483 (25.5%) | 1357 (25.8%) | 126 (22.3%) |  |
| 75-79 | 1288 (22.1%) | 1151 (21.9%) | 137 (24.2%) |  |
| 80-84 | 1075 (18.4%) | 942 (17.9%) | 133 (23.5%) |  |
| 85-90 | 627 (10.7%) | 561 (10.6%) | 66 (11.7%) |  |
| 90+ | 255 (4.3%) | 218 (4.1%) | 37 (6.5%) |  |
| Female sex | 3216 (55.4%) | 2886 (54.9%) | 330 (58.4%) | 0.128 |
| **General health information** |  |  |  |  |
| Overall health condition |  |  |  | <.001* |
| Excellent | 793 (13.6%) | 740 (14.1%) | 53 (9.3%) |  |
| Very good | 1926 (33.1%) | 1775 (33.8%) | 151 (26.7%) |  |
| Good | 1990 (34.2%) | 1804 (34.4%) | 186 (32.9%) |  |
| Fair | 907 (15.5%) | 769 (14.6%) | 138 (24.4%) |  |
| Poor | 200 (3.3%) | 163 (3.1%) | 37 (6.5%) |  |
| Heart attack^1^ | 121 (2.0%) | 105 (1.9%) | 16 (2.8%) | 0.240 |
| Heart disease^1^ | 1050 (18.0%) | 916 (17.4%) | 134 (23.7%) | <.001* |
| High blood pressure^1^ | 3977 (68.5%) | 3565 (67.9%) | 412 (72.9%) | 0.016* |
| Arthritis^1^ | 3440 (59.2%) | 3054 (58.2%) | 386 (68.3%) | <.001* |
| Osteoporosis^1^ | 1303 (22.3%) | 1166 (22.2%) | 137 (24.2%) | 0.300 |
| Diabetes^1^ | 1518 (26.0%) | 1338 (25.5%) | 180 (31.8%) | 0.001* |
| Lung disease^1^ | 962 (16.5%) | 833 (15.9%) | 129 (22.8%) | <.001* |
| Stroke^1^ | 106 (1.7%) | 89 (1.7%) | 17 (3.0%) | 0.040* |
| Dementia or alzheimers^1^ | 139 (2.3%) | 121 (2.3%) | 18 (3.1%) | 0.246 |
| Cancer^1^ | 330 (5.6%) | 292 (5.5%) | 38 (6.7%) | 0.297 |
| Broken or fractured bones^1^ | 241 (4.0%) | 203 (3.8%) | 38 (3.9%) | 0.002* |
| Overnight hospital stay^1^ | 1166 (20.0%) | 1021 (19.44%) | 145 (25.7%) | <.001* |
| Previous surgery^1^ | 721 (12.3%) | 636 (12.1%) | 85 (15.0%) | 0.05 |
| BMI [Mean (STD)] | 33.1 (7.6) | 33.4 (7.7) | 33.1 (7.5) | 0.424 |
| Under a normal BMI | 34 (0.4%) | 29 (0.5%) | 5 (0.9%) | 0.489 |
| Over the normal BMI | 5193 (89.5%) | 4694 (89.4%) | 499 (88.3%) | 0.476 |
| **Info. adjacent to health** |  |  |  |  |
| Fell in the last month | 0 (0.0%) | 0 (0.0%) | 0 (0.0%) | 1.000 |
| Worried about falling^2^ | 1512 (26.0%) | 1282 (24.4%) | 230 (40.7%) | <.001* |
| Fell in the last year | 1402 (24.0%) | 1176 (22.4%) | 226 (40.0%) | <.001* |
| Depressive, anxious, or unsatisfied traits^2^ |  |  |  | <.001* |
| Not at all | 2784 (47.9%) | 2577 (49.0%) | 207 (36.6%) |  |
| Several days | 2016 (34.8%) | 1804 (34.3%) | 212 (37.5%) |  |
| More than half the days | 484 (34.9%) | 410 (7.8%) | 74 (13.1%) |  |
| Nearly every day | 532 (9.0%) | 460 (8.8%) | 72 (12.7%) |  |
| Trouble sleeping^2^ |  |  |  | 0.310 |
| Never | 828 (14.2%) | 751 (14.3%) | 77 (13.6%) |  |
| Rarely (once a week or less) | 1478 (25.4%) | 1358 (25.9%) | 120 (21.2%) |  |
| Some nights (2-4 a week) | 1694 (29.1%) | 1535 (29.2%) | 159 (28.1%) |  |
| Most nights1 (5-6 a week) | 691 (11.8%) | 616 (11.7%) | 75 (13.3%) |  |
| Every night | 1125 (19.3%) | 991 (18.9%) | 134 (23.7%) |  |
| Lost 10 pounds^1^ | 1470 (25.2%) | 1284 (24.4%) | 186 (32.9%) | <.001* |
| **Information on home situation**  situation |  |  |  |  |
| Total number in household [Mean(std)] | 1.98 (1.02) | 2.09 (1.15) | 1.97 (1.02) | 0.006* |
| Number of children (stepchildren)[Mean(std)] | 3.25 (2.45) | 3.32 (2.46) | 3.24 (2.45) | 0.448 |
| Ramp at entrance of home | 506 (8.6%) | 446 (8.4%) | 60 (10.6%) | 0.104 |
| Strong community links |  |  |  | 0.800 |
| Do not agree | 160 (2.7%) | 142 (2.7%) | 18 (3.2%) |  |
| Agree a little | 1194 (20.5%) | 1079 (20.5%) | 115 (20.3%) |  |
| Agree a lot | 4462 (76.9%) | 4030 (76.7%) | 432 (76.4%) |  |
| **Information on mobility / general ability** |  |  |  |  |
| Uses a cane, walker, wheelchair, or scooter^2^ | 1234 (21.2%) | 1036 (19.7%) | 198 (35.0%) | <.001* |
| Hearing problems | 1268 (21.8%) | 1107 (21.1%) | 161 (28.4%) | <.001* |
| Vision problems | 5462 (94.1%) | 4939 (94.0%) | 523 (92.6%) | 0.188 |
| Problems with chewing or swallowing^2^ | 440 (7.5%) | 370 (7.0%) | 70 (12.4%) | <.001* |
| Have problems speaking^2^ | 198 (3.3%) | 162 (3.1%) | 36 (6.4%) | <.001* |
| Bothered by pain^2^ | 3055 (52.6%) | 2705 (51.5%) | 350 (61.9%) | <.001* |
| Use medication for pain^2^ |  |  |  | <.001* |
| Never | 2084 (35.9%) | 1897 (36.1%) | 187 (33.1%) |  |
| Rarely (once a week or less) | 1438 (24.7%) | 1330 (25.3%) | 108 (19.1%) |  |
| Some days (2-4 a week) | 1023 (17.5%) | 912 (17.4%) | 111 (19.6%) |  |
| Most days (5-6 a week) | 289 (4.9%) | 249 (4.7%) | 40 (7.1%) |  |
| Every day | 982 (16.8%) | 863 (16.4%) | 119 (21.1%) |  |
| Have breathing problems^2^ | 1070 (18.4%) | 931 (17.7%) | 139 (24.6%) | <.001* |
| Limited strength in the body^2^ | 2514 (43.2%) | 2181 (41.5%) | 333 (58.9%) | <.001* |
| Low energy^2^ | 2421 (41.7%) | 2117 840.3%) | 304 (53.8%) | <.001* |
| Balance and coordination problems^2^ | 1528 (26.3%) | 1278 (24.3%) | 250 (44.2%) | <.001* |
| Able to walk 6 blocks^2^ | 4030 (69.4%) | 3722 (71.0%) | 308 (54.5%) | <.001* |
| Can walk up 20 stairs^2^ | 4462 (76.9%) | 4105 (78.2%) | 357 (63.2%) | <.001* |
| Can carry 20 pounds^2^ | 4434 (76.4%) | 4066 (77.4%) | 368 (65.1%) | <.001* |
| Able to get on knees and back up^2^ | 2697 (33.5%) | 2507 (47.7%) | 190 (33.6%) | <.001* |
| Able to put a heavy object on a shelf^2^ | 4979 (85.8%) | 4547 (86.6%) | 432 (76.5%) | <.001* |
| Can open a jar with one hand^2^ | 4531 (78.0%) | 4136 (78.8%) | 395 (69.9%) | <.001* |
| Able to recall a portion of the date | 5725 (98.7%) | 5172 (98.5%) | 553 (97.9%) | 0.342 |
| State of memory compared to a year ago |  |  |  | 0.130 |
| Much worse | 35 (0.5%) | 29 (0.5%) | 6 (1.1%) |  |
| Worse | 596 (10.2%) | 524 (10.0%) | 72 (12.7%) |  |
| Same | 4948 (85.3%) | 4486 (85.4%) | 462 (81.8%) |  |
| Better | 185 (3.1%) | 166 (3.2%) | 19 (3.4%) |  |
| Much better | 52 (0.9%) | 46 (1.0%) | 6 (1.1%) |  |
| Used help to get outside^2^ | 258 (4.3%) | 211 (4.0%) | 47 (8.3%) | <.001* |
| How often goes outside^2^ |  |  |  | 0.037* |
| Never | 0 (0.0%) | 0 (0.0%) | 0 (0.0%) |  |
| Rarely (once a week or less) | 109 (1.8%) | 95 (1.8%) | 14 (2.5%) |  |
| Some days (2-4 a week) | 529 (9.0%) | 462 (8.8%) | 67 (11.8%) |  |
| Most days (5-6 a week) | 1008 (17.3%) | 923 (17.6%) | 85 (15.0%) |  |
| Every day | 4170 (71.9%) | 3771 (71.8%) | 399 (70.6%) |  |
| Difficulty in going outside^2^ |  |  |  | <.001* |
| None | 5182 (89.3%) | 4741 (90.3%) | 441 (78%) |  |
| A little | 406 (6.9%) | 327 (6.2%) | 79 (14.0%) |  |
| Some | 181 (3.0%) | 147 (2.3%) | 34 (6.0%) |  |
| A lot | 47 (0.7%) | 36 (0.7%) | 11 (2.0%) |  |
| How often leaves their building vs a year ago |  |  |  | <.001* |
| Less often | 578 (9.9%) | 495 (9.4%) | 83 (14.7%) |  |
| About the same | 4762 (82.1%) | 4319 (82.2%) | 443 (78.4%) |  |
| More often | 476 (8.1%) | 437 (8.3%) | 39 (7.0%) |  |
| How often holds onto walls or furniture^2^ |  |  |  | <.001* |
| Never | 3242 (55.8%) | 3014 (57.4%) | 228 (40.3%) |  |
| Rarely | 1298 (22.3%) | 1167 (22.2%) | 131 (23.2%) |  |
| Sometimes | 911 (15.6%) | 774 (14.7%) | 137 (24.2%) |  |
| Most times | 229 (3.8%) | 194 (3.7%) | 35 (6.2%) |  |
| Every time | 136 (2.2%) | 102 (1.9%) | 34 (6.0%) |  |
| Difficulty getting around inside the house^2^ |  |  |  | <.001* |
| None | 5157 (88.9%) | 4713 (89.7%) | 444 (78.6%) |  |
| A little | 459 (7.8%) | 378 (7.2%) | 81 (14.3%) |  |
| Some | 184 (3.1%) | 149 (2.8%) | 35 (6.2%) |  |
| A lot | 16 (0.2%) | 11 (0.2%) | 5 (0.9%) |  |
| Difficulty getting out of bed^2^ |  |  |  | <.001* |
| None | 5039 (86.9%) | 4588 (87.4%) | 451 (78.8%) |  |
| A little | 518 (8.8%) | 443 (8.4%) | 75 (13.3%) |  |
| Some | 219 (3.7%) | 188 (3.5%) | 31 (5.5%) |  |
| A lot | 40 (0.6%) | 32 (0.6%) | 8 (1.4%) |  |
| **Ability to perform daily activities**  activities |  |  |  |  |
| How often gets dressed vs a year ago |  |  |  | 0.056 |
| Less often | 99 (1.6%) | 84 (1.6%) | 15 2 (6.0%) |  |
| Same | 5584 (96.2%) | 5052 (96.2%) | 532 (94.1%) |  |
| More often | 133 (2.2%) | 115 (2.1%) | 18 (3.2%) |  |
| Difficulty in washing up^2^ |  |  |  | <.001* |
| None | 5353 (92.3%) | 4860 (92.5%) | 493 (87.2%) |  |
| A little | 336 (5.6%) | 284 (5.4%) | 52 (9.2%) |  |
| Some | 104 (1.7%) | 86 (1.6%) | 18 (3.1%) |  |
| A lot | 23 (0.3%) | 21 (0.4%) | 2 (0.3%) |  |
| How often wash up compared to last year |  |  |  | 0.129 |
| Less often | 200 (3.3%) | 174 (3.3%) | 26 (4.6%) |  |
| Same | 5391 (92.9%) | 4879 (92.9%) | 512 (90.6%) |  |
| More often | 225 (3.8%) | 198 (3.7%) | 27 (4.8%) |  |
| Uses tools to aid in toilet use | 1318 (22.6%) | 1139 (21.7%) | 179 (31.7%) | <.001* |
| Difficulty using the toilet^2^ |  |  |  | <.001* |
| None | 5598 (96.5%) | 5078 (96.7%) | 520 (92%) |  |
| A little | 153 (2.5%) | 118 (2.2%) | 35 (6.2%) |  |
| Some | 55 (0.7%) | 49 (1.0%) | 6 (1.1%) |  |
| A lot | 10 (0.1%) | 6 (0.1%) | 4 (0.7%) |  |
| Needs help using the toilet | 20 (0.2%) | 14 (0.2%) | 6 (1.0%) | 0.007* |
| Has a regular doctor | 5551 (95.7%) | 5011 (95.4%) | 540 (95.6%) | 0.959 |
| Has seen a regular doctor in the last year | 5435 (93.7%) | 4896 (93.2%) | 539 (95.4%) | 0.060 |
| **NHATS performance**  activities |  |  |  |  |
| Grip score |  |  |  | 0.027* |
| 0 (worst) | 463 (7.9%) | 411 (7.8%) | 52 (9.2%) |  |
| 1 | 1256 (21.6%) | 1120 (21.3%) | 136 (24.1%) |  |
| 2 | 1398 (24%) | 1260 (24%) | 138 (24.4%) |  |
| 3 | 1409 (24.2%) | 1266 (24.1%) | 143 (25.3%) |  |
| 4 (best) | 1290 (22.1%) | 1194 (22.7%) | 96 (17.0%) |  |
| Balance score |  |  |  | <.001* |
| 0 (worst) | 271 (4.6%) | 234 (4.4%) | 37 (6.5%) |  |
| 1 | 1126 (19.3%) | 972 (18.5%) | 154 (27.2%) |  |
| 2 | 1608 (27.6%) | 1433 (27.3%) | 175 (31.0%) |  |
| 3 | 1453 (25.0%) | 1321 (25.1%) | 132 (23.3%) |  |
| 4 (best) | 1358 (23.3%) | 1291 (24.6%) | 67 (11.9%) |  |
| Walking score |  |  |  | <.001* |
| 0 (worst) | 201 (3.4%) | 173 (3.3%) | 28 (5.0%) |  |
| 1 | 1399 (24.0%) | 1217 (23.2%) | 182 (32.2%) |  |
| 2 | 1508 (25.9%) | 1354 (25.8%) | 102 (27.3%) |  |
| 3 | 1377 (23.7%) | 1275 (24.3%) | 154 (18.0%) |  |
| 4 (best) | 1331 (22.9%) | 1232 (23.5%) | 99 (17.5%) |  |
| Chair score |  |  |  | <.001* |
| 0 (worst) | 830 (14.2%) | 718 (13.7%) | 112 (19.8%) |  |
| 1 | 1198 (20.6%) | 1061 (20.2%) | 137 (24.2%) |  |
| 2 | 1227 (21.1%) | 1112 (21.2%) | 115 (20.3%) |  |
| 3 | 1349 (23.2%) | 1241 (23.6%) | 108 (19.1%) |  |
| 4 (best) | 1212 (20.8%) | 1119 (21.3%) | 93 (16.4%) |  |

For each participant, answers were recorded in one year, as well as the following. The responses in the first year were analyzed, and the model predicted the answer to a question in the following year: "Have you fallen in the last month?", in essence predicting falls one year in advance during the specific month in which the following round of interviews was conducted. When observing population characteristics, the total population is observed, as well as differences between the participants who had reported falling during the next round of interviews and those who had not.

*P<0.05

1. The question refers to the time period of the year prior to the interview.
2. The question refers to the time period of the month prior to the interview.


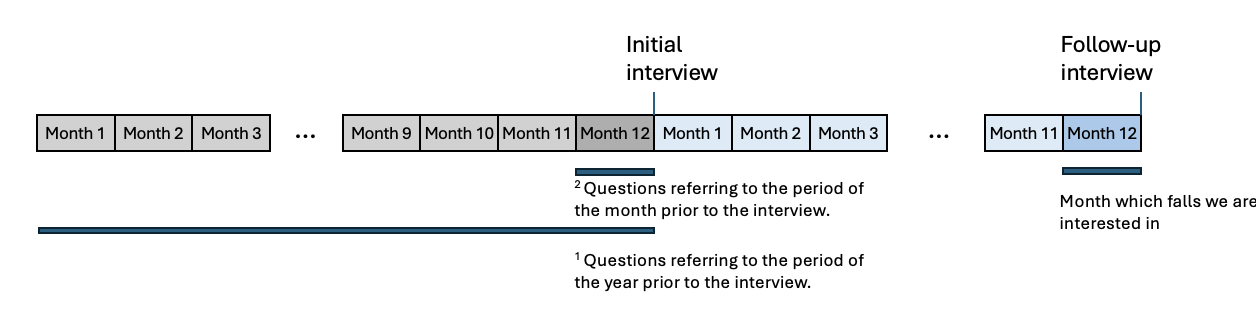


Supplementary Figure 1: Graphical representation of the interview timeline. The 1 and 2 superscripts refer to those describing features in Tables 1-3 and Supp Table 1.

Supplementary Table 2: Number of participants initially interviewed each year incorporated to form the dataset.

| Year | 2012 | 2013 | 2014 | 2015 | 2016 | 2017 | 2018 | 2019 | 2021 | 2022 | Total |
| --- | --- | --- | --- | --- | --- | --- | --- | --- | --- | --- | --- |
| Number of participants initially interviewed | 2790 | 520 | 158 | 102 | 1708 | 337 | 112 | 55 | 13 | 21 | 5816 |

Performance activities were not recorded in 2020.

Supplementary Table 3: Category comparisons for Age variable

| Risk factor category comparison | P |
| --- | --- |
| Age (years) |  |
| 65-69 vs 70-74 | 0.025 |
| 70-74 vs 75-79 | 0.064 |
| 75-79 vs 80-84 | 0.209 |
| 80-84 vs 85-90 | 0.287 |
| 85-90 vs 90+ | 0.120 |

Supplementary Table 4: Category comparisons for the Health information variables

| Risk factor category comparison | P |
| --- | --- |
| Overall health |  |
| Excellent vs Very good | 0.337 |
| Very good vs Good | 0.104 |
| Good vs Fair | <.001* |
| Fair vs Poor | 0.296 |
| Use medication for pain^2^ |  |
| Never vs Rarely | 0.139 |
| Rarely vs Some days | 0.005 |
| Some days vs Most days | 0.193 |
| Most days vs Every day | 0.498 |
| Nervous, anxious or unsatisfied traits^2^ |  |
| Not at all vs Several days | <.001* |
| Several days vs More than half of the days | 0.008 |
| More than half of the days vs Nearly every day | 0.133 |

Supplementary Table 5: Category comparisons for the Mobility information variables

| Risk factor category comparison | P |
| --- | --- |
| How often leaves the building compared to a year ago |  |
| Less often vs About the same | <.001* |
| About the same vs More often | 0.474 |
| How often holds onto walls or furniture |  |
| Never vs Rarely | <.001* |
| Rarely vs Sometimes | <.001* |
| Sometimes vs Most times | 1.000 |
| Most times vs Every time | 0.031 |

## Supplementary Table 6: Category comparisons for the Difficulty information variables

| Risk factor category comparison | P |
| --- | --- |
| Difficulty using the toilet^2^ |  |
| None vs A little | <.001* |
| A little vs Some | 0.086 |
| Some vs A lot | 0.062 |
| Difficulty getting out of bed^2^ |  |
| None vs A little | <.001* |
| A little vs Some | 1.000 |
| Some vs A lot | 0.478 |
| Difficulty in washing up^2^ |  |
| None vs A little | <.001* |
| A little vs Some | 0.77 |
| Some vs A lot | 0.478 |
| Difficulty getting around inside the house ^2^ |  |
| None vs A little | <.001* |
| A little vs Some | 0.767 |
| Some vs A lot | 0.397 |
| Difficulty in going outside^2^ |  |
| None vs A little | <.001* |
| A little vs Some | 0.938 |
| Some vs A lot | 0.615 |

Supplementary Table 7: Category comparisons for the NHATS activities scores variables

| Risk factor category comparison | P |
| --- | --- |
| Grip score |  |
| 0 (worst) vs 1 | 0.880 |
| 1 vs 2 | 0.456 |
| 2 vs 3 | 0.855 |
| 3 vs 4 (best) | 0.161 |
| Balance score |  |
| 0 (worst) vs 1 | 1.000 |
| 1 vs 2 | 0.031* |
| 2 vs 3 | 0.111 |
| 3 vs 4 (best) | <.001* |
| Walking score |  |
| 0 (worst) vs 1 | 0.803 |
| 1 vs 2 | 0.021* |
| 2 vs 3 | 0.010* |
| 3 vs 4 (best) | 1.000 |
| Chair score |  |
| 0 (worst) vs 1 | 0.187 |
| 1 vs 2 | 0.110 |
| 2 vs 3 | 0.245 |
| 3 vs 4 (best) | 0.811 |

A

Supplementary Table 8: OR and confidence intervals for Logistic Regression with individual features.

| Risk factor | OR | CI (95%) |
| --- | --- | --- |
| **Demographic data** |  |  |
| Age (years) |  |  |
| 65-69* |  |  |
| 70-74 | 1.44 | 1.06-1.96 |
| 75-79 | 1.84 | 1.36-2.50 |
| 80-84 | 2.19 | 1.61-2.98 |
| 85-90 | 1.82 | 1.28-2.60 |
| 90+ | 2.63 | 1.71-4.03 |
| Female sex | 1.15 | 0.97-1.37 |
| **General health information** |  |  |
| Overall health condition |  |  |
| Excellent |  |  |
| Very good | 1.19 | 0.86-1.64 |
| Good | 1.44 | 1.05-1.98 |
| Fair | 2.51 | 1.80-3.49 |
| Poor | 3.17 | 2.02-4.98 |
| Heart attack^1^ | 1.43 | 0.84-2.43 |
| Heart disease^1^ | 1.47 | 1.20-1.81 |
| High blood pressure^1^ | 1.27 | 1.05-1.55 |
| Arthritis^1^ | 1.55 | 1.29-1.87 |
| Osteoporosis^1^ | 1.12 | 0.91-1.37 |
| Diabetes^1^ | 1.37 | 1.13-1.65 |
| Lung disease^1^ | 1.57 | 1.27-1.94 |
| Stroke^1^ | 1.80 | 1.06-3.05 |
| Dementia or alzheimers^1^ | 1.40 | 0.84-2.31 |
| Cancer^1^ | 1.22 | 0.86-1.74 |
| Broken or fractured bones^1^ | 1.79 | 1.25-2.56 |
| Overnight hospital stay^1^ | 1.43 | 1.17-1.75 |
| Previous surgery^1^ | 1.29 | 1.01-1.64 |
| BMI | 1.00 | 0.99-1.02 |
| Under a normal BMI | 1.61 | 0.62-4.17 |
| Over the normal BMI | 0.90 | 0.68-1.18 |
| **Info. adjacent to health** |  |  |
| Fell in the last month | / | / |
| Worried about falling^2^ | 2.13 | 1.78-2.54 |
| Fell in the last year | 2.31 | 1.93-2.77 |
| Depressive, anxious, or unsatisfied traits^2^ |  |  |
| Not at all |  |  |
| Several days | 1.46 | 1.20-1.79 |
| More than half the days | 2.25 | 1.69-2.99 |
| Nearly every day | 1.95 | 1.46-2.59 |
| Trouble sleeping^2^ |  |  |
| Never |  |  |
| Rarely (once a week or less) | 0.86 | 0.64-1.16 |
| Some nights (2-4 a week) | 1.01 | 0.76-1.34 |
| Most nights1 (5-6 a week) | 1.19 | 0.85-1.66 |
| Every night | 1.32 | 0.98-1.77 |
| Lost 10 pounds^1^ | 1.52 | 1.26-1.83 |
| **Information on home situation**  situation |  |  |
| Total number in household | 1.11 | 1.03-1.20 |
| Number of children (stepchildren) | 1.01 | 0.98-1.05 |
| Ramp at entrance of home | 1.28 | 0.97-1.70 |
| Strong community links |  |  |
| Do not agree |  |  |
| Agree a little | 0.84 | 0.50-1.42 |
| Agree a lot | 0.85 | 0.51-1.39 |
| **Information on mobility / general ability** |  |  |
| Uses a cane, walker, wheelchair, or scooter^2^ | 2.20 | 1.82-2.64 |
| Hearing problems | 1.49 | 1.23-1.81 |
| Vision problems | 0.79 | 0.56-1.10 |
| Problems with chewing or swallowing^2^ | 1.87 | 1.42-2.45 |
| Have problems speaking^2^ | 2.14 | 1.47-3.10 |
| Bothered by pain^2^ | 1.53 | 1.28-1.83 |
| Use medication for pain^2^ |  |  |
| Never |  |  |
| Rarely (once a week or less) | 0.82 | 0.64-1.05 |
| Some days (2-4 a week) | 1.23 | 0.96-1.58 |
| Most days (5-6 a week) | 1.63 | 1.13-2.35 |
| Every day | 1.40 | 1.10-1.78 |
| Have breathing problems^2^ | 1.51 | 1.23-1.86 |
| Limited strength in the body^2^ | 2.02 | 1.69-2.41 |
| Low energy^2^ | 1.72 | 1.45-2.05 |
| Balance and coordination problems^2^ | 2.47 | 2.07-2.95 |
| Able to walk 6 blocks^2^ | 0.49 | 0.41-0.59 |
| Can walk up 20 stairs^2^ | 0.48 | 0.40-5 0.58 |
| Can carry 20 pounds^2^ | 0.54 | 0.45-0.66 |
| Able to get on knees and back up^2^ | 0.55 | 0.46-0.67 |
| Able to put a heavy object on a shelf^2^ | 0.50 | 0.41-0.62 |
| Can open a jar with one hand^2^ | 0.63 | 0.52-0.76 |
| Able to recall a portion of the date | 0.70 | 0.38-1.30 |
| State of memory compared to a year ago |  |  |
| Much worse |  |  |
| Worse | 0.66 | 0.27-1.65 |
| Same | 0.50 | 0.21-1.21 |
| Better | 0.55 | 0.20-1.50 |
| Much better | 0.63 | 0.19-2.14 |
| Used help to get outside^2^ | 2.17 | 1.56-3.01 |
| How often goes outside^2^ |  |  |
| Never | / | / |
| Rarely (once a week or less) |  |  |
| Some days (2-4 a week) | 0.98 | 0.53-1.82 |
| Most days (5-6 a week) | 0.62 | 0.34-1.14 |
| Every day | 0.72 | 0.41-1.27 |
| Difficulty in going outside^2^ |  |  |
| None |  |  |
| A little | 2.60 | 1.99-3.38 |
| Some | 2.49 | 1.69-3.66 |
| A lot | 3.28 | 1.66-6.50 |
| How often leaves their building vs a year ago |  |  |
| Less often |  |  |
| About the same | 0.61 | 0.48-0.79 |
| More often | 0.53 | 0.36-0.80 |
| How often holds onto walls or furniture^2^ |  |  |
| Never |  |  |
| Rarely | 1.48 | 1.18-1.86 |
| Sometimes | 2.34 | 1.87-2.93 |
| Most times | 2.38 | 1.62-3.50 |
| Every time | 4.41 | 2.92-6.65 |
| Difficulty getting around inside the house^2^ |  |  |
| None |  |  |
| A little | 2.27 | 1.76-2.95 |
| Some | 2.49 | 1.71-3.65 |
| A lot | 4.82 | 1.67-13.95 |
| Difficulty getting out of bed^2^ |  |  |
| None |  |  |
| A little | 1.72 | 1.33-2.24 |
| Some | 1.68 | 1.13-2.48 |
| A lot | 2.54 | 1.16-5.55 |
| **Ability to perform daily activities**  activities |  |  |
| How often gets dressed vs a year ago |  |  |
| Less often |  |  |
| Same | 0.59 | 0.34-1.03 |
| More often | 0.88 | 0.42-1.84 |
| Difficulty in washing up^2^ |  |  |
| None |  |  |
| A little | 1.80 | 1.32-2.46 |
| Some | 2.06 | 1.23-3.46 |
| A lot | 0.94 | 0.22-4.016 |
| How often wash up compared to last year |  |  |
| Less often |  |  |
| Same | 0.70 | 0.46-1.07 |
| More often | 0.91 | 0.51-1.62 |
| Uses tools to aid in toilet use | 1.67 | 1.39-2.02 |
| Difficulty using the toilet^2^ |  |  |
| None |  |  |
| A little | 2.90 | 1.97-4.27 |
| Some | 1.20 | 0.51-2.80 |
| A lot | 6.51 | 1.83-23.14 |
| Needs help using the toilet | 4.02 | 1.54-10.49 |
| Has a regular doctor | 1.03 | 0.68-1.58 |
| Has seen a regular doctor in the last year | 1.50 | 1.00-2.26 |
| **NHATS performance**  activities |  |  |
| Grip score |  |  |
| 0 (worst) |  |  |
| 1 | 0.96 | 0.68-1.35 |
| 2 | 0.87 | 0.62-1.21 |
| 3 | 0.89 | 0.64-1.25 |
| 4 (best) | 0.64 | 0.45-0.91 |
| Balance score |  |  |
| 0 (worst) |  |  |
| 1 | 1.00 | 0.68-1.47 |
| 2 | 0.77 | 0.53-1.13 |
| 3 | 0.63 | 0.43-0.93 |
| 4 (best) | 0.33 | 0.21-0.50 |
| Walking score |  |  |
| 0 (worst) |  |  |
| 1 | 0.92 | 0.60-1.42 |
| 2 | 0.70 | 0.46-1.08 |
| 3 | 0.49 | 0.32-0.77 |
| 4 (best) | 0.50 | 0.32-0.78 |
| Chair score |  |  |
| 0 (worst) |  |  |
| 1 | 0.83 | 0.64-1.08 |
| 2 | 0.63 | 0.50-0.88 |
| 3 | 0.56 | 0.42-0.74 |
| 4 (best) | 0.53 | 0.40-0.71 |

*Because one category is considered as a reference by the model (automatically the first one), it has no coefficient as it has been absorbed by the intercept. Each CI is thus for the odds ratio of being in that category versus the reference.

Supplementary Table 9: Significant risk factors in the Logistic Regression with Lasso regularization associated with falls during the month 11 months after the initial interview

| Risk factor | OR | CI 95% |
| --- | --- | --- |
| Demographic data | | |
| Age | 1.09 | 1.00-1.21 |
| General health information | | |
| BMI | 0.97 | 0.96-1.00 |
| Information adjacent to health | | |
| Fallen in the past year | 1.76 | 1.43- 2.08 |
| Information on home situation | | |
| Total number in household | 1.05 | 1.03-1.19 |
| Information on mobility and general ability | | |
| Balance and coordination problems | 1.41 | 1.04-1.73 |
| How often goes outside | 1.18 | 1.05-1.39 |
| How often holds onto walls/furniture | 1.10 | 1.00-1.21 |
| Scores on NHATS activities | | |
| Chair score | 1.00 | 1.00-1.20 |


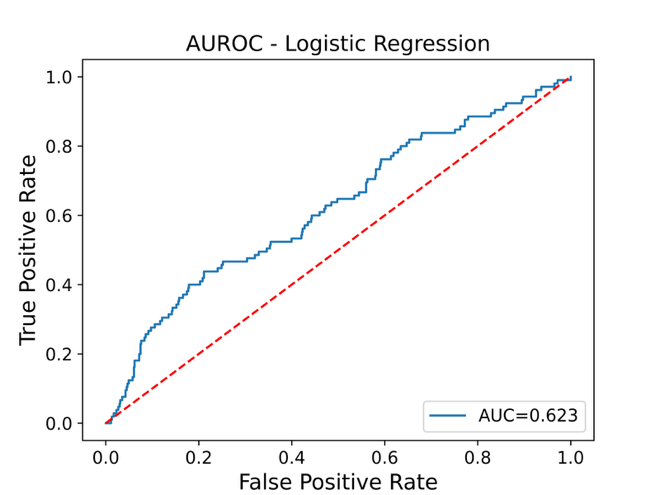

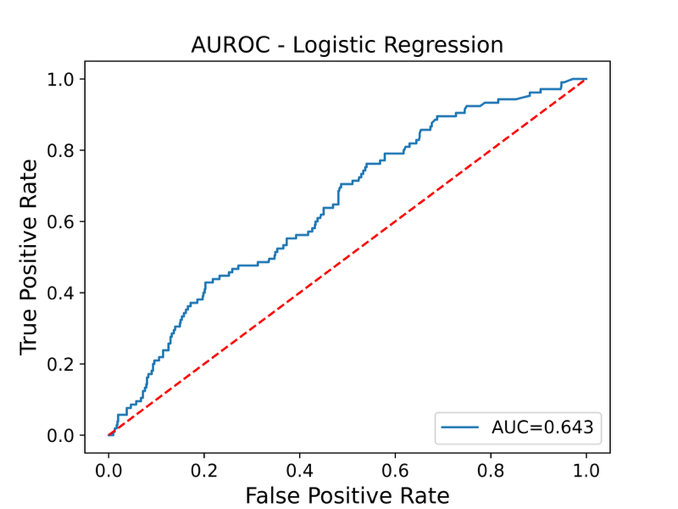


Supplementary Figure 2: AUROC for full (left) and reduced (right) logistic regression models. Models were trained on 5000 instances and validated on 816.
